# Association Between Disease Activity of Systemic Lupus Erythematosus and Resting Electrocardiogram Abnormalities

**DOI:** 10.3390/jcm14061799

**Published:** 2025-03-07

**Authors:** Lin Wu, Changlin Zhao, Jingjing Chen, Li Xu, Xianguan Yu, Xinghua Guo, Zhiming Lin, Xiaoying Xie, Bin Zhou, Yong Liu

**Affiliations:** 1 Department of Cardiology, The Third Affiliated Hospital, Sun Yat-sen University, Guangzhou 510630, China; wulin23@mail.sysu.edu.cn (L.W.); zhaoclzssy@126.com (C.Z.); xuli20220528@163.com (L.X.); yuxg3@mail.sysu.edu.cn (X.Y.); xiexy227@mai2.sysu.edu.cn (X.X.); 2 Big Data and Artifcial Intelligence Center, The Third Afliated Hospital of Sun Yat-sen University, Guangzhou 510630, China; chenjj336@mail.sysu.edu.cn; 3 Department of Rheumatology, The Third Affiliated Hospital, Sun Yat-sen University, Guangzhou 510630, China; xinghuaguo@foxmail.com (X.G.); lzm-zj99@163.com (Z.L.)

**Keywords:** systemic lupus erythematosus, electrocardiogram, ST-T changes, machine learning model

## Abstract

**Objective:** The association between the activity of SLE and abnormalities of ECG remains not well elucidated. We aimed to examine the relationship between the SLE Disease Activity Index 2000 (SLEDAI-2K) and abnormalities of ECG in a Chinese population. **Methods:** Data for this cross-sectional study were retrieved from an SLE database (2018–2023). According to the SLEDAI-2K, patients were categorized into inactive, mild activity, moderate activity, and severe activity groups. Weighted multivariable regression analyses and subgroup analyses were conducted to assess the independent relationship between the SLEDAI-2K and ECG abnormalities. Restricted cubic splines (RCSs) were employed to explore potential non-linear correlations. **Results:** A total of 317 SLE patients (282 women; mean age 30.0 [23.0; 43.0]) were included. The overall prevalence of ST segment changes and T wave abnormalities was 37.5%. Our findings indicated a linear relationship between the SLEDAI-2K and the risk of ST-T changes. We used interaction terms to assess heterogeneity among subgroups and discovered significant differences specifically related to female gender, age (≤25 years), combined autoimmune diseases, and infectious complications. This suggested that the positive association between the SLEDAI-2K and ST-T changes was influenced by participants’ gender, age, presence of combined autoimmune diseases, and infectious complications. **Conclusions:** Higher SLEDAI-2K scores were associated with an increased incidence of ST-T changes in SLE patients. The SLEDAI-2K is anticipated to emerge as an effective index for identifying early heart involvement in this population.

## 1. Introduction

Systemic lupus erythematosus (SLE) is an autoimmune disease that involves multiple internal organs, including the heart. Cardiac involvement in SLE can affect the pericardium, myocardium, valvular apparatus, and coronary arteries, contributing to morbidity and mortality in this disease [1]. Previous studies have revealed that, compared with the general population, SLE patients have a more than 2-fold increased risk of cardiovascular disease [2]. Additionally, several types of arrhythmias such as atrioventricular block, right bundle branch block, left anterior fascicular block, and sick sinus syndrome resulting from immune-mediated damage to the conduction tissue have been observed in SLE patients [3]. During active inflammation, sinus tachycardia is most common in SLE patients [1]. Currently, ECG is a non-invasive and convenient tool to screen for heart disease, including ischemic heart disease, arrhythmias, myocardial abnormalities, and structural heart disease [3]. ECG abnormalities indicate ongoing damage in the conduction system and cardiomyocytes.

Previous studies have reported that 14.8% to 18% of SLE patients had chronic tachycardia [1,4]. Atrial fibrillation was present in 0.13% to 3% of SLE patients [1,5]. QT prolongation is a high-prevalence phenomenon in SLE patients (15.3% to 17%) [1,5]. Our SLE patient cohort revealed a high prevalence of ST segment changes and T wave changes, which indicate myocardial injury [6].

Assessing disease activity in systemic lupus erythematosus (SLE) is essential for evaluating patient outcomes, identifying differences among SLE subgroups, monitoring responses to proposed new medications, and conducting longitudinal disease assessments in observational studies and clinical trials [7]. The SLEDAI-2K is an effective tool to evaluate the complex multisystem nature of this disease with fluctuating levels of disease activity [8]. At present, the SLEDAI-2K is the most widely used disease activity index in clinical practice and trials [9]. However, the association between the SLEDAI-2K and ECG abnormalities remains essentially unknown, and few large-scale studies have investigated the relationship between the SLEDAI-2K and the risk of ECG abnormalities in the Han Chinese population. Therefore, we sought to determine if the SLEDAI-2K held prognostic value for ECG abnormalities in patients with SLE through the present single-center interdisciplinary study.

## 2. Methods

### 2.1. Study Design

In this cross-sectional study, consecutive records of 317 SLE patients were collected from the database of the Division of Rheumatology of the Third Affiliated Hospital of Sun Yat-sen University from August 2018 to December 2023. All patients were diagnosed with SLE based on the 1997 American College of Rheumatology (ACR) criteria. The inclusion criteria were an age ≥ 18 years and having undergone a 12-lead resting ECG during their inpatient stay within the above-mentioned period. The exclusion criteria were (1) history of a CVD event (angina, myocardial infarction, congestive heart failure, angioplasty, and pacemaker) or severe valvular disease; (2) the presence of hepatic failure, renal failure, overlapping syndrome, and/or abnormal serum electrolytes; (3) pregnancy; (4) malignant tumors; (5) thyroid disorders; (6) right bundle branch block/left bundle branch block or right ventricular hypertrophy/left ventricular hypertrophy; and (7) excessive noise in their ECG data or incomplete baseline data.

The diagnoses of lupus nephritis (LN) and secondary Sjögren syndrome (sSS) were confirmed either using the 2016 ACR criteria or by conducting biopsies on the patients’ kidneys and labial glands. The diagnosis of neuropsychiatric SLE (NPSLE) was based on clinical evaluation, serological studies, and imaging results [6]. The diagnosis of SLE-associated interstitial lung disease (ILD) was based on persistent symptoms and high-resolution computed tomography (HR-CT), according to the definition of idiopathic interstitial pneumonia by the Japanese Ministry of Health and Welfare [7].

This study complied with the Declaration of Helsinki. All patients provided informed consent and the study was approved by the Ethics Committee of the Third Affiliated Hospital, Sun Yat-sen University.

**Figure 1:**
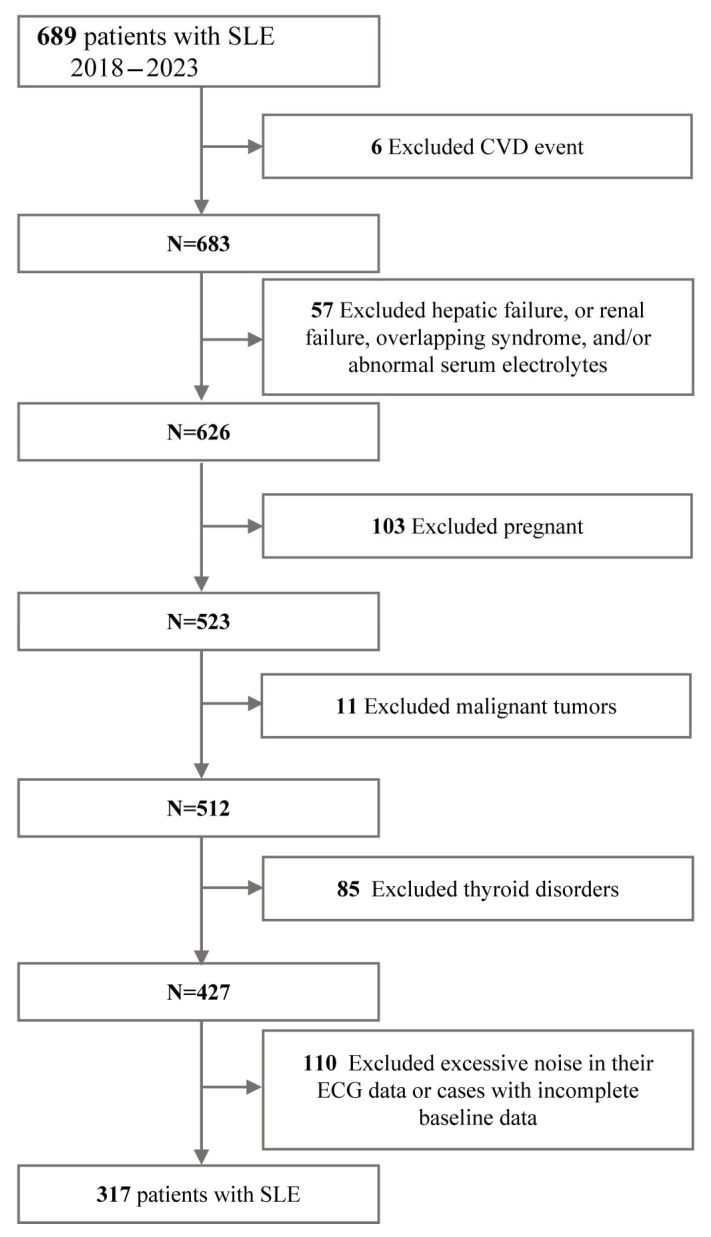
Flowchart of the sample selection from the SLE dataset.

The flowchart of the patient recruitment process is shown in Figure 1.

### 2.2. Data Processing

A standard protocol including demographics, complications, assessment of disease activity (SLE Disease Activity Index 2000; SLEDAI-2K), laboratory tests, and standard 12-lead resting ECG reports was used to collect data. The ECG data were acquired by an internist (paper speed: 25 mm/s; calibration: 1 mV = 10 mm). The raw ECG data were stored in XML format (ECGNET Vision 3.0; SanRui Electronic Technology, Guangdong, China). Standardized 10s 12-lead ECG signals were collected at a sampling rate of 1000 Hz. In total, 180 ECG features were automatically extracted by the ECG system. The 12-lead ECGs were analyzed by two cardiologists with more than 10 years of experience.

### 2.3. Assessment of the Activity and Complications of SLE

According to the SLEDAI-2K, patients were categorized into an inactive group (0–4 score), a mild activity group (5–9 score), a moderate activity group (10–14 score), and a severe activity group (>14 score).

The patients with SLE were categorized into Group 1 (patients without complications or comorbidities), Group 2 (patients with LN), Group 3 (patients with neuropsychiatric involvement), Group 4 (patients with hematological involvement), Group 5 (patients with infection), and Group 6 (patients with other complications such as sSS, hematological involvement, musculoskeletal symptoms, ankylosing spondylitis (AS), and lupus mesenteric vasculitis). The groups were chosen as the exposure variable.

#### 2.3.1. Assessment of ECG Abnormalities

ECGs were categorized as normal or abnormal. ECG abnormalities were recorded as (1) sinus arrhythmia, (2) atrial hypertrophy and/or ventricular hypertrophy, (3) atrial arrhythmia and/or ventricular arrhythmia, (4) ST segment and/or T wave abnormalities, and (5) other ECG abnormalities.

Sinus arrhythmia included sinus tachycardia [heart rate (HR) ≥ 100 beats per minute (bpm) on ECG] and sinus bradycardia [heart rate (HR)\<60 beats per minute (bpm) on ECG]. Atrial arrhythmia included atrial premature beat, atrial tachycardia, atrial flutter, and atrial fibrillation. Ventricular arrhythmia included ventricular premature beat, ventricular tachycardia, ventricular flutter, and ventricular fibrillation. Atrioventricular (AV) block included first- to third-degree AV block. Other ECG abnormalities included (1) left axis deviation and/or right axis deviation, (2) left bundle branch block (LBBB), (3) right bundle branch block (RBBB), and (4) QT interval prolongation. QT interval prolongation was defined as a corrected QT-c ≥ 450 ms. When more than one abnormality was observed in the same patient, each was recorded separately.

ST segment abnormalities were defined as a horizontal or downward tilt 0.5 mm or greater from the baseline, quantified at 80 ms outside the J point. T wave abnormalities included notched, asymmetric, flat-wave (<1 mm amplitude of the T wave peak), flat-peak (>1 mm amplitude of the T wave peak), and broad (width ≥ 2 × the height of the T wave).

#### 2.3.2. Assessment of Covariates

Information on various demographic and health-related factors was collected, including gender, age at time of SLE diagnosis (≤25 vs. >25 years), disease duration, cumulative SLE manifestations (neuropsychiatric involvement), LN, hematological involvement, infectious complications, combined autoimmune diseases, serum creatinine, serum complement C3, serum complement C4, anti-nuclear antibodies (ANA), anti-double-stranded DNA, anti-Smith antibodies, and anti-SSB antibodies.

#### 2.3.3. Statistical Analysis

Study participants were classified into four groups according to the SLEDAI-2K. Continuous variables were expressed as the mean ± standard deviation (SD) or the median and interquartile range (IQR), while categorical variables were presented as numbers (percentage). Differences in the descriptive analyses were assessed using a one-way ANOVA for continuous variables and chi-squared tests for categorical variables. Multivariable logistic regression was utilized to assess the correlation between the SLEDAI-2K and ECG abnormalities using three distinct models for statistical inference. Model 1 was unadjusted. Model 2 was adjusted for age and gender. Model 3 was adjusted for gender, age at time of SLE diagnosis (≤25 vs. >25 years), disease duration, cumulative SLE manifestations (neuropsychiatric involvement), LN, hematological involvement, infectious complications, combined autoimmune diseases, serum creatinine, serum complement C3, serum complement C4, ANA, anti-double-stranded DNA, anti-Smith antibodies, and anti-SSB antibodies. In the sensitivity analyses, the SLEDAI-2K was categorized into four groups to assess the robustness of the results, and the likelihood of ECG abnormalities across these groups was evaluated. Additionally, we used restricted cubic spline (RCS) analyses with three piecewise points to explore potential relationships between the SLEDAI-2K and the likelihood of ECG abnormalities. For the subgroup analysis, the data were stratified by gender (male/female), age (≤25/>25 years), infectious complications (yes/no), and combined autoimmune diseases (yes/no). Statistical significance was defined as a two-sided *p*-value < 0.05. The statistical analysis was performed using R software, version 4.4.1 (Institute for Statistics and Mathematics, Vienna, Austria; www.r-project.org).

## 3. Results

### 3.1. Clinical Characteristics

Table 1 shows the baseline characteristics of the study participants stratified by disease activity according to the SLEDAI-2K. From January 2018 to December 2023, a total of 317 participants were included in our study, with an average age of 30.0 [23.0; 43.0] years and 282 (89.0%) of them were female. According to the SLEDAI-2K, the patients were divided into an inactive group (138 cases, 118 (85.5%) females, and 33.0 [24.5; 45.8]), a mild activity group (96 cases, 89 (92.7%) females, and 30.0 [24.0; 40.0]), a moderate activity group (49 cases, 46 (93.9%) females, and 30.0 [21.0; 40.0]), and a severe activity group (34 cases, 29 (85.3%) females, and 25.5 [22.0; 34.8]). The average SLEDAI-2K was 6.00 [3.00; 10.0], and the SLEDAI-2K values for each group were 2.00 [0.00; 4.00], 7.00 [6.00; 8.00], 12.0 [10.0; 12.0], and 18.0 [16.0; 20.8], respectively.

Among the study group, 51 patients had atrial arrhythmia, 51 patients had atrial premature beats, 3 patients had atrial tachycardia, and 5 patients had ventricular arrhythmia.

The overall incidence of ST-T changes was 119 (37.5%). Participants with a higher SLEDAI-2K demonstrated a higher rate of ST-T changes (inactive group: 35 (25.4%); mild activity group: 46 (47.9%); moderate activity group: 16 (32.7%); severe activity group: 22 (64.7%); *p* < 0.001). As the level of SLE activity increased, the risk of developing atrial arrhythmia and ventricular arrhythmia tended to increase (inactive group: 14 (10.1%); mild activity group: 26 (27.1%); moderate activity group: 10 (20.4%); severe activity group: 6 (17.6%); *p* = 0.009).

Participants with a higher SLEDAI-2K were more likely to have neuropsychiatric SLE (inactive group: 4 (2.90%); mild activity group: 2 (2.08%); moderate activity group: 8 (16.3%); severe activity group: 10 (29.4%); *p* < 0.001), lupus nephritis (inactive group: 21 (15.2%); mild activity group: 28 (29.2%); moderate activity group: 27 (55.1%); severe activity group: 23 (67.6%); *p* < 0.001), and concomitant autoimmune diseases (inactive group: 20 (14.5%); mild activity group: 14 (14.6%); moderate activity group: 1 (2.04%); severe activity group: 6 (17.6%); *p* = 0.049). Moreover, significant differences in biochemical indicators and immunological markers were observed between the groups, with participants in the highest quartile exhibiting significantly higher levels of serum creatinine, lower levels of complement C3 and complement C4, and higher levels of anti-dsDNA antibodies.

Our findings revealed significant differences between participants with and without complications or comorbidities. Compared with participants without complications or comorbidities, those with complications or comorbidities had higher levels of SLEDAI-2K, serum creatinine, anti-nuclear antibodies, and anti-dsDNA antibodies and lower levels of complement C3 and complement C4 (Supplementary Table S1). There was an increasing trend between a higher SLEDAI-2K and the likelihood of sinus arrhythmia, first-degree atrioventricular block, and ST-T changes, although this trend was not statistically significant.

### 3.2. Relationship Between the SLEDAI-2K and the Likelihood of ECG Abnormalities

The association between the SLEDAI-2K and the risk of ECG abnormalities is shown in Table 2 and Table 3, and Supplementary Tables S2–S4. For the risk of ST-T changes, this positive association remained stable (OR = 1.10, 95%CI: 1.04–1.17, and *p* < 0.001) in the fully adjusted model (Model 3). In addition, we performed a sensitivity analysis by converting the SLEDAI-2K from a continuous variable into a categorical variable (disease activity groups). The multivariable-adjusted ORs and 95%CIs from the lowest to the highest SLE disease activity groups were 1.00 (reference), 3.11 (1.71; 5.75), 1.54 (0.67; 3.51), and 7.82 (2.82; 23.33), respectively.

There was no significant association between the SLEDAI-2K and the likelihood of sinus arrhythmia or other ECG abnormalities in our study (Supplementary Tables S2 and S4).

Table 3 shows that the mild activity group was associated with increased odds of atrial arrhythmia and ventricular arrhythmia in all three models (Model 1: OR = 3.29, 95%CI: (1.64; 6.87), and *p* = 0.001; Model 2: 3.51 (1.72; 7.43), and *p* < 0.001; Model 3: 3.36 (1.59; 7.43), and *p* = 0.002).

Supplementary Table S3 shows an increasing trend between a higher SLEDAI-2K and the likelihood of atrioventricular block, although this trend was not statistically significant.

### 3.3. RCS Analysis

**Figure 2:**
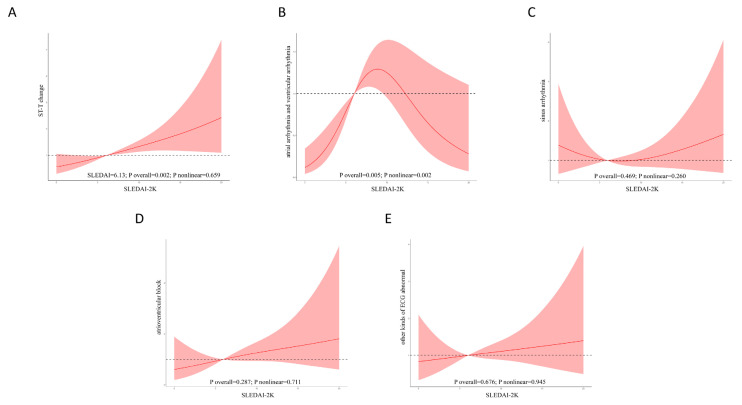
The restricted cubic spline (RCS) analyses of the SLEDAI-2K and the different types of ECG abnormalities. (**A**) The restricted cubic spline (RCS) analysis of the SLEDAI-2K and the risk of ST-T changes. (**B**) The restricted cubic spline (RCS) analysis of the SLEDAI-2K and the risk of atrial arrhythmia and ventricular arrhythmia. (**C**) The restricted cubic spline (RCS) analysis of the SLEDAI-2K and the risk of sinus arrhythmia. (**D**) The restricted cubic spline (RCS) analysis of the SLEDAI-2K and the risk of atrioventricular block. (**E**) The restricted cubic spline (RCS) analysis of the SLEDAI-2K and the risk of other types of ECG abnormalities.

We used restricted cubic spline (RCS) curves to examine the potential linearity in the relationship between the SLEDAI-2K and the likelihood of ST-T changes, as illustrated in Figure 2A. Our findings suggested a weak linear association (*p* for non-linearity = 0.659). Below a threshold of 6.13 in the SLEDAI-2K, we observed a strongly increasing relationship between the SLEDAI-2K and ST-T change likelihood (overall *p* = 0.002). To the right of the inflection point at 6.13, we noted a significantly steeper upward trend in the relationship between the SLEDAI-2K and ST-T changes.

In addition, our results indicated an approximately linear relationship between the SLEDAI-2K and the likelihood of atrial arrhythmia and ventricular arrhythmia, sinus arrhythmia, atrioventricular block, and other ECG abnormalities (Figure 2B–E).

### 3.4. Subgroup Analysis

Subgroup analyses and interaction tests were used to analyze the consistency of the relationship between the SLEDAI-2K and ST-T changes, atrial arrhythmia and ventricular arrhythmia, sinus arrhythmia, and other ECG abnormalities across various population subgroups. These subgroups included gender, age (>25 years or ≤25 years), concomitant autoimmune diseases, and infectious complications.

**Figure 3:**
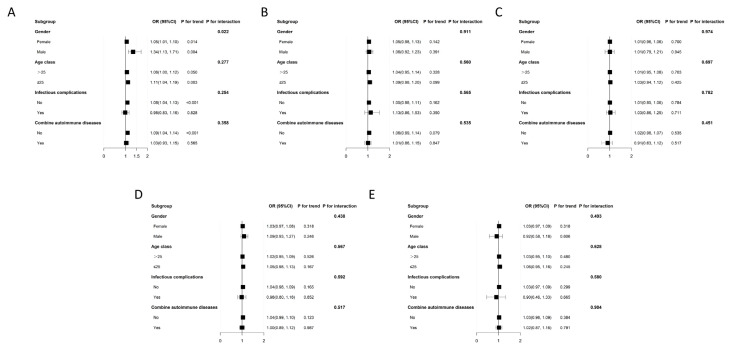
Subgroup analyses of the association between the SLEDAI-2K and the risk of ST-T changes, atrial arrhythmia and ventricular arrhythmia, sinus arrhythmia, atrioventricular block, and other types of ECG abnormalities. (**A**) Subgroup analysis of the association between the SLEDAI-2K and the risk of ST-T changes. (**B**) Subgroup analysis of the association between the SLEDAI-2K and the risk of atrial arrhythmia and ventricular arrhythmia. (**C**) Subgroup analysis of the association between the SLEDAI-2K and the risk of sinus arrhythmia. (**D**) Subgroup analysis of the association between the SLEDAI-2K and the risk of atrioventricular block. (**E**) Subgroup analysis of the association between the SLEDAI-2K and other types of ECG abnormalities.

The risk of ST-T changes was higher in male patients (OR = 1.34, 95%CI: 1.13–1.71, and *p* = 0.004), those aged ≤ 25 years (OR = 1.11, 95%CI: 1.04–1.19, and *p* = 0.003), patients without infectious complications (OR = 1.08, 95%CI: 1.04–1.13, and *p* < 0.001), and those without concomitant autoimmune diseases (OR = 1.09, 95%CI: 1.04–1.14, and *p* < 0.001) (Figure 3A; Supplementary Table S5).

For the likelihood of atrial arrhythmia and ventricular arrhythmia (Figure 3B; Supplementary Table S6), sinus arrhythmia (Figure 3C; Supplementary Table S7), atrioventricular block (Figure 3D; Supplementary Table S8), and other ECG abnormalities (Figure 3E; Supplementary Table S9), no statistically significant interaction was detected (*p* > 0.05), indicating that there was no dependence on gender, age, concomitant autoimmune diseases, or infectious complications for these associations.

### 3.5. Association Between ST-T Changes, Atrial Arrhythmia and Ventricular Arrhythmia, Sinus Arrhythmia, Atrioventricular Block, and Other ECG Abnormalities and the SLEDAI-2K

We analyzed changes in the SLEDAI-2K in individuals with ECG abnormalities and different types of ECG abnormalities (Supplementary Table S10). In the fully adjusted model (Model 3), we observed an increase in the SLEDAI-2K of 0.09 units in individuals with ST-T changes (β: 0.09; 95%CI: 0.04–0.15) compared with participants without ST-T changes. In contrast, no significant change in the SLEDAI-2K was observed in atrial arrhythmia and ventricular arrhythmia, sinus arrhythmia, atrioventricular block, or other ECG abnormalities.

## 4. Discussion

In our cross-sectional study of 317 patients with systemic lupus erythematosus (SLE), the most prevalent electrocardiogram (ECG) abnormalities were ST segment and/or T wave changes (37.5%). Moreover, we found that a higher SLE Disease Activity Index 2000 (SLEDAI-2K) score was independently associated with an increased risk of ST-T changes. The association between the SLEDAI-2K and the likelihood of ST-T changes, sinus arrhythmia, atrioventricular block, and other ECG abnormalities exhibited a linear relationship. There were non-linear associations between the SLEDAI-2K and atrial arrhythmia and ventricular arrhythmia, although the trends did not reach statistical significance. Our study demonstrated that the SLEDAI-2K could serve as an effective predictor of ECG abnormalities. We found an increase in the SLEDAI-2K of 0.09 units in individuals with ST-T changes (β: 0.09; 95%CI: 0.04–0.15) compared with participants without ST-T changes. The risk of ST-T changes was higher in male patients and those aged ≤25 years. The prevalence of ECG abnormalities in SLE patients was consistent with previous studies [6].

The SLEDAI-2K offers several advantages in assessing disease activity in patients with SLE. It evaluates disease activity across multiple organ systems affected by SLE, providing a comprehensive view of the patient’s condition [10]. The SLEDAI-2K is sensitive to both improvements and deteriorations in disease activity, making it effective for monitoring treatment responses over time [7]. As a standardized tool, it allows for consistent assessments across different clinicians and research studies, facilitating comparisons and collaborations [11]. The British Isles Lupus Assessment Group Index (BILAG) is used to assess disease activity of individual organs, while the SLEDAI-2K is an effective and reliable tool to determine global improvement [12]. Compared with the European Consensus Lupus Activity Measurement (ECLAM) and the Revised Systemic Lupus Activity Measure (SLAM-R), the SLEDAI-2K and BILAG are sensitive to both improvements and deteriorations in disease activity, making them effective for monitoring treatment responses over time [13]. BILAG contains 101 items and 5 additional items, while the SLEDAI-2K consists of 24 items covering 9 organ systems. The SLEDAI-2K is convenient and time-saving, and is widely used in randomized clinical trials (RCTs) and outcome assessments [9,14]. By encompassing these advantages, the SLEDAI-2K serves as a valuable tool for clinicians and researchers in the management and study of SLE.

Non-specific ST and T wave abnormalities refer to subtle changes such as mild or up-sloping ST segment depressions and flat or slightly inverted T waves. These are some of the most frequent ECG findings, with an estimated prevalence of 4–10% in asymptomatic subjects. Numerous epidemiological studies have demonstrated a link between isolated non-specific ST and T wave abnormalities and cardiovascular disease (CVD) mortality independent of traditional coronary risk factors [15]. The prevalence of ST-T changes in patients with SLE has been reported in previous studies. Riette du Toit et al. found that non-specific ST segment and T wave abnormalities (70%) were most frequently observed in patients with lupus myocarditis [16]. Anca D Askanase et al. reported that 44% of patients with SLE had non-specific ST segment and T wave abnormalities [17]. Our previous study showed that T wave changes (52.3%) and non-specific ST-T changes (26.6%) were the most prevalent abnormalities among SLE patients with abnormal ECGs [6]. There are other common causes of ST-T abnormalities on a resting ECG, particularly hyperventilation, mitral valve prolapse, and concave-shaped chest wall conformation. In a previous study, positive exercise test results were observed in 45.4% of subjects with an ST decline induced by hyperventilation but only 13.1% of subjects had T wave abnormalities [18]. In addition, non-specific ST-T wave abnormalities in inferior leads were detected in 28.1% (range of 0–73%) of mitral valve prolapse individuals [19], which may represent a variable combination of metabolic, ischemic, or myopathic disorders [20]. In our current study, we found that ST-T changes (37.5%) were the most prevalent ECG abnormalities in SLE patients.

The correlation between the SLEDAI-2K and the likelihood of ST-T changes has been rarely reported in previous studies. Hypertension, ANA titer, disease severity (SLEDAI-2K), age, and disease duration were associated with ST-T changes [6]. Older age was associated with the presence of non-specific ST-T abnormalities. There was no association between the SLEDAI-2K, clinical data, and the risk of ST-T abnormalities [5,17]. Murray B Urowitz et al. found that myocardial perfusion defects were strongly and independently predictive of coronary artery disease (CAD), although disease activity was not associated with abnormalities on myocardial perfusion imaging [21]. In our study, as the level of SLE activity increased, the risk of ST-T changes also increased. Restricted cubic spline (RCS) analyses determined a weak linearity in the relationship between the SLEDAI-2K and the likelihood of ST-T changes. For SLEDAI-2K scores higher than 6.13, we observed a strong increasing relationship between the SLEDAI-2K score and the likelihood of ST-T changes (overall *p* = 0.002). Subgroup analyses demonstrated that being male (OR = 1.34), aged ≤25 years (OR = 1.11), without infectious complications (OR = 1.08), and without concurrent autoimmune diseases (OR = 1.09) were associated with an elevated likelihood of ST-T changes. This indicated that higher SLEDAI-2K scores were associated with a higher probability of experiencing ST-T changes on an ECG. After a multivariable adjustment for traditional CVD risk factors and clinical data, the presence of ST-T changes remained significantly associated with the extent of SLE disease activity. The multivariable-adjusted odds ratio (OR) of the severe activity group was 7.82 compared with the inactivity group. Patients with SLE are at an increased risk of developing atherosclerotic CVD compared with individuals without SLE [22]. Although the clinical significance of non-specific ST-T changes in SLE patients without CVD is unclear, it is tempting to speculate that these changes may represent subclinical CVD [17].

In our study, SLE patients had a lower prevalence of sinus arrhythmia, including sinus tachycardia and sinus bradycardia (16.1%). Michael H. Weisman et al. reported a higher prevalence of sinus tachycardia (18%) [1], while Utset et al. found a prevalence of 14.8% [4]. Although the association between sinus arrhythmia and the SLEDAI-2K did not reach statistical significance, there was a weak linear relationship between sinus arrhythmia and the SLEDAI-2K. Different disease activities, combined autoimmune diseases, or other immune diseases may cause sinus tachycardia. However, our study showed no statistically significant differences in the above states.

SLE can affect the heart, including its conduction system, although the involvement of the conduction system is less frequently reported. An atrioventricular block is indicative of myocardial conduction system injury, which occurs in the absence of significant cardiac disease and is generally attributed to idiopathic fibrosis of the conduction system [23]. In our study, all atrioventricular blocks were first-degree. This observation aligned with the established understanding that a higher-degree atrioventricular (AV) block, such as a complete heart block, is an extremely rare manifestation of SLE [3,24]. A previous study reported the prevalence of a first-degree AV block to be 3% in SLE patients [1] and 0.95% had a complete atrioventricular block. K Tselios et al. found that 0.95% of SLE patients had a complete atrioventricular block. Anekwe E. Onwuanyi reported a 45-year-old asymptomatic female with SLE who had an alternating first- and second-degree AV block, which was relieved after treatment with glucocorticoids [25]. In our study, 5% (16 of 317) of SLE patients had a first-degree AV block. There was no association between disease activity and AV block.

Atrial arrhythmia and ventricular arrhythmia occurred in 16.1% and 1.58% of SLE patients, respectively. The prevalence of ventricular arrhythmia was similar to the 1.40% reported by Christian A et al., while the prevalence of atrial arrhythmia was much higher than the 1.15% they reported [5]. In our study, mild activity was closely associated with atrial arrhythmia and ventricular arrhythmia after adjusting for all variables.

In our study, 25 (7.89%) patients presented with other ECG abnormalities such as QT interval prolongation, left axis deviation, right axis deviation, left bundle branch block, or right bundle branch block. QT prolongation is widely acknowledged to be a significant factor in cardiac morbidity as it is directly linked to Torsades de Pointes (TdP). The proportion of our SLE patients with QT interval prolongation (2.5%) was lower than the 7.3% reported by Bourré-Tessier J et al. [26] and the 3.7% found in our previous research [6]. Tarik Demir et al. found that the QTc was similar in SLE and control groups [1], but the QT dispersion (QTd) and corrected QT dispersion were significantly increased in SLE patients compared with controls [5,27]. The QTd may sensitively indicate silent myocardial involvement in SLE patients [28]. In patients with SLE, the QTd was significantly higher among those with high disease activity (SLEDAI > 10) compared with those with mild to moderate (SLEDAI ≤ 10) disease activity [29]. Lazzerini et al. identified a correlation between prolonged QT intervals and the presence of anti-SSA/Ro antibodies [30]. Our study revealed a potential linear relationship between the SLEDAI-2K and other ECG abnormalities, including prolonged QT intervals, axis deviation, and bundle branch block.

We unveiled that neuropsychiatric SLE, lupus nephritis, and combined other autoimmune diseases were consistently important factors for disease activity. As disease activity intensifies, serum creatinine and anti-dsDNA levels increase, while complement C3 and complement C4 levels decrease. Elevated serum creatinine indicates severe renal involvement in patients with high SLE activity, elevated anti-dsDNA levels, and decreased inflammatory activity in complement C3 and complement C4, which affect cardiac rhythm and conduction function. The complement is a crucial component of humoral autoimmunity and inflammation plays a key role in atherosclerosis, which may further affect non-specific ST-T changes [31].

In brief, we mitigated a confounding bias by adjusting for numerous covariates, thereby enhancing the reliability of our results and extending their applicability to a broader range of individuals. Additionally, we conducted sensitivity analyses and employed subgroup analysis models to further strengthen the reliability of our assessment regarding the association between the SLEDAI-2K and the likelihood of ECG abnormalities. As a non-invasive, convenient, and affordable tool, an ECG may be recommended for all SLE patients during their initial and follow-up visits because managing this systemic and chronic disease requires interdisciplinary cooperation to comprehensively assess the condition over time.

Although our research had advantages, there were also potential limitations that should not be ignored. Firstly, although we adjusted for as many potential covariates as possible, there was still a possibility of residual confounding. Secondly, our analysis mainly involved participants from one country, which may have limited the generalizability and applicability of our research results globally due to the varying prevalence of SLE. Thirdly, our sample size was relatively limited. Fourthly, due to our use of resting 12-lead ECGs instead of Holter monitoring, a large amount of patient ECG information could not be collected. Fifthly, cardiac-related examinations such as echocardiography and magnetic resonance imaging were not applied in this study. Therefore, we could not clearly explain the causes of the ECG abnormalities, particularly the ST-T changes, nor could we identify any correlation between cardiac injury or inflammation and the activity of SLE. In the near future, we plan to conduct a comparative evaluation of ECG and cardiac MRI findings in SLE patients. Sixthly, variations in patient disease course and adjustments in medication regimens meant that the use of glucocorticoids, immunosuppressants, and other treatments was not fully documented and thus their potential effects on abnormal electrocardiograms were not evaluated in this study. We plan to address this limitation in future research.

## 5. Conclusions

In conclusion, our data revealed that ST-T changes were the most common abnormalities observed in the ECGs of SLE patients. The SLEDAI-2K served as a valuable tool for assessing the likelihood of abnormal ECG development in SLE patients and the relationship between the SLEDAI-2K and ST-T changes was non-linear. In addition, our research has the potential to reduce screening costs. These findings are particularly relevant to clinical practice and extensive epidemiological research.

## Figures and Tables

**Table 1:** Baseline characteristics of the study population.

Activity of Disease	All Participants	Inactive	Mild Activity	Moderate Activity	Severe Activity	*p*-Value
N	317	138	96	49	34	
SLEDAI	6.00 [3.00; 10.0]	2.00 [0.00; 4.00]	7.00 [6.00; 8.00]	12.0 [10.0; 12.0]	18.0 [16.0; 20.8]	<0.001
Gender						0.192
Female (N, %)	282 (89.0%)	118 (85.5%)	89 (92.7%)	46 (93.9%)	29 (85.3%)	
Male (N, %)	35 (11.0%)	20 (14.5%)	7 (7.29%)	3 (6.12%)	5 (14.7%)	
Age (Year)	30.0 [23.0; 43.0]	33.0 [24.5; 45.8]	30.0 [24.0; 40.0]	30.0 [21.0; 40.0]	25.5 [22.0; 34.8]	0.021
Age Class						0.045
>25 (N, %)	217 (68.5%)	103 (74.6%)	65 (67.7%)	32 (65.3%)	17 (50.0%)	
≤25 (N, %)	100 (31.5%)	35 (25.4%)	31 (32.3%)	17 (34.7%)	17 (50.0%)	
Disease Duration (Year)	2.00 [0.00; 6.00]	2.00 [0.00; 6.00]	1.00 [0.00; 6.00]	2.00 [0.00; 8.00]	1.50 [0.00; 4.00]	0.566
Neuropsychiatric SLE						<0.001
No (N, %)	293 (92.4%)	134 (97.1%)	94 (97.9%)	41 (83.7%)	24 (70.6%)	
Yes (N, %)	24 (7.57%)	4 (2.90%)	2 (2.08%)	8 (16.3%)	10 (29.4%)	
Lupus Nephritis						<0.001
No (N, %)	218 (68.8%)	117 (84.8%)	68 (70.8%)	22 (44.9%)	11 (32.4%)	
Yes (N, %)	99 (31.2%)	21 (15.2%)	28 (29.2%)	27 (55.1%)	23 (67.6%)	
Interstitial Lung Disease						0.747
No (N, %)	300 (94.6%)	132 (95.7%)	89 (92.7%)	46 (93.9%)	33 (97.1%)	
Yes (N, %)	17 (5.36%)	6 (4.35%)	7 (7.29%)	3 (6.12%)	1 (2.94%)	
Hematological Involvement						0.789
No (N, %)	293 (92.4%)	129 (93.5%)	89 (92.7%)	44 (89.8%)	31 (91.2%)	
Yes (N, %)	24 (7.57%)	9 (6.52%)	7 (7.29%)	5 (10.2%)	3 (8.82%)	
Combined Autoimmune Diseases						0.049
No (N, %)	276 (87.1%)	118 (85.5%)	82 (85.4%)	48 (98.0%)	28 (82.4%)	
Yes (N, %)	41 (12.9%)	20 (14.5%)	14 (14.6%)	1 (2.04%)	6 (17.6%)	
Infectious Complications						0.364
No (N, %)	293 (92.4%)	131 (94.9%)	88 (91.7%)	43 (87.8%)	31 (91.2%)	
Yes (N, %)	24 (7.57%)	7 (5.07%)	8 (8.33%)	6 (12.2%)	3 (8.82%)	
Sinus Arrhythmia						0.263
No (N, %)	266 (83.9%)	116 (84.1%)	81 (84.4%)	44 (89.8%)	25 (73.5%)	
Yes (N, %)	51 (16.1%)	22 (15.9%)	15 (15.6%)	5 (10.2%)	9 (26.5%)	
Atrial Arrhythmia and Ventricular Arrhythmia						**0.009**
No (N, %)	261 (82.3%)	124 (89.9%)	70 (72.9%)	39 (79.6%)	28 (82.4%)	
Yes (N, %)	56 (17.7%)	14 (10.1%)	26 (27.1%)	10 (20.4%)	6 (17.6%)	
ST-T Changes						**<0.001**
No (N, %)	198 (62.5%)	103 (74.6%)	50 (52.1%)	33 (67.3%)	12 (35.3%)	
Yes (N, %)	119 (37.5%)	35 (25.4%)	46 (47.9%)	16 (32.7%)	22 (64.7%)	
Other Types of ECG Abnormality						0.081
No (N, %)	292 (92.1%)	128 (92.8%)	92 (95.8%)	44 (89.8%)	28 (82.4%)	
Yes (N, %)	25 (7.89%)	10 (7.25%)	4 (4.17%)	5 (10.2%)	6 (17.6%)	
Serum creatinine	58.0 [49.0; 74.0]	57.0 [49.1; 68.8]	54.3 [47.6; 67.5]	62.0 [49.0; 90.0]	85.4 [69.1; 137]	**<0.001**
Complement C3 (g/L)	0.71 [0.47; 0.91]	0.81 [0.68; 1.02]	0.71 [0.48; 0.89]	0.58 [0.37; 0.76]	0.40 [0.25; 0.54]	**<0.001**
Complement C4 (g/L)	0.15 [0.07; 0.20]	0.16 [0.12; 0.22]	0.14 [0.07; 0.19]	0.09 [0.04; 0.18]	0.06 [0.03; 0.15]	**<0.001**
Anti-Nuclear Antibodies (+) (N, %)	103 (32.5%)	38 (27.5%)	37 (38.5%)	17 (34.7%)	11 (32.3%)	0.106
Anti-dsDNA Antibodies (+) (N, %)	24 (7.6%)	13 (9.4%)	2 (2.0%)	15 (30.6%)	6 (17.6%)	**0.010**
Anti-RNP Antibody (+) (N, %)	121 (38.2%)	51 (37.0%)	40 (41.7%)	15 (30.6%)	15 (44.1%)	0.751
Anti-Smith Antibodies (+) (N, %)	94 (29.7%)	34 (24.7%)	33 (34.3%)	14 (28.6%)	13 (28.2%)	0.912
Anti-SSB Antibodies (+) (N, %)	63 (19.8%)	30 (21.7%)	21 (21.8%)	12 (21.9%)	6 (17.6%)	0.420
Anti-Jo-1 Antibody (+) (N, %)	7 (5.4%)	12 (8.7%)	3 (3.1%)	0 (0%)	2 (5.9%)	0.084
Anti-Scl-70 Antibody (+) (N, %)	20 (6.3%)	13 (9.4%)	4 (4.2%)	1 (2.0)	2 (5.9%)	0.104
Anti-SSA Antibody (+) (N, %)	164 (51.7%)	64 (46.3%)	60 (62.5%)	23 (46.9%)	17 (50%)	0.804

Bold indicates statistical significance. A one-way ANOVA was utilized for continuous variables and the chi-squared test was employed for categorical variables to assess differences in the descriptive analyses.

**Table 2:** The association between the SLEDAI-2K and the risk of ST-T changes.

ST-T Change	OR (95%CI)		
	Model 1	Model 2	Model 3
SLEDAI (continuous)	1.08 (1.03; 1.12)	1.08 (1.04; 1.13)	1.1 (1.04; 1.17)
	***p* < 0.001**	***p* < 0.001**	***p* < 0.001**
SLEDAI (classification)			
Inactive	Reference	Reference	Reference
Mild activity	2.71 (1.56; 4.74)	2.82 (1.61; 4.99)	3.11 (1.71; 5.75)
	***p* < 0.001**	***p* < 0.001**	***p* < 0.001**
Moderate activity	1.43 (0.69; 2.88)	1.52 (0.73; 3.1)	1.54 (0.67; 3.51)
	*p* = 0.326	*p* = 0.258	*p* = 0.306
Severe activity	5.4 (2.46; 12.35)	6.21 (2.78; 14.55)	7.82 (2.82; 23.33)
	***p* < 0.001**	***p* < 0.001**	***p* < 0.001**

Bold indicates statistical significance. Model 1: no covariates were adjusted. Model 2: age and gender were adjusted. Model 3: gender, age (≤25 vs. >25 years), disease duration, cumulative SLE manifestations (neuropsychiatric involvement), LN, hematological involvement, infectious complications, combined autoimmune diseases, serum creatinine, serum complement C3, serum complement C4, ANA, anti-double-stranded DNA, anti-Smith antibodies, and anti-SSB antibodies were adjusted. OR: odds ratio 95%CI: 95% confidence interval.

**Table 3:** The association between the SLEDAI-2K and the risk of atrial arrhythmia and ventricular arrhythmia.

Atrial Arrhythmia and Ventricular Arrhythmia	OR (95%CI)		
	Model 1	Model 2	Model 3
SLEDAI (continuous)	1.03 (0.98; 1.08)	1.04 (0.99; 1.10)	1.03 (0.97; 1.1)
	*p* = 0.180	*p* = 0.094	*p* = 0.365
SLEDAI (classification)			
Inactive	Reference	Reference	Reference
Mild activity	3.29 (1.64; 6.87)	3.51 (1.72; 7.43)	3.36 (1.59; 7.43)
	***p* = 0.001**	***p* < 0.001**	***p* = 0.002**
Moderate activity	2.27 (0.91; 5.49)	2.5 (0.99; 6.19)	2.51 (0.88; 7.03)
	*p* = 0.070	***p* = 0.047**	*p* = 0.080
Severe activity	1.90 (0.63; 5.19)	2.32 (0.75; 6.56)	1.76 (0.46; 6.25)
	*p* = 0.227	*p* = 0.122	*p* = 0.389

Bold indicates statistical significance. Atrial arrhythmia included atrial premature beat, atrial tachycardia, atrial flutter, and atrial fibrillation. Ventricular arrhythmia included ventricular premature beat, ventricular tachycardia, ventricular flutter, and ventricular fibrillation. Model 1: no covariates were adjusted. Model 2: age and gender were adjusted. Model 3: gender, age (≤25 vs. >25 years), disease duration, cumulative SLE manifestations (neuropsychiatric involvement), LN, hematological involvement, infectious complications, combined autoimmune diseases, serum creatinine, serum complement C3, serum complement C4, ANA, anti-double-stranded DNA, anti-Smith antibodies, and anti-SSB antibodies were adjusted. OR: odds ratio; 95%CI: 95% confidence interval.

## Data Availability

The original contributions presented in this study are included in the article/Supplementary Materials. Further inquiries can be directed to the corresponding author(s).

## Supplementary Materials

The following supporting information can be downloaded at: https://www.mdpi.com/article/10.3390/jcm14061799/s1, Table S1: Baseline characteristics of the study population by with or without complications or complications; Table S2: The association between SLEDAI-2K and the risk of sinus arrhythmia; Table S3:The association between SLEDAI-2K and the risk of atrioventricular block; Table S4: The association between SLEDAI-2K and the risk of other kinds of ECG abnormal; Table S5: The association of ST-T change on SLEDAI-2K levels in different subgroup; Table S6: The association of atrial arrhythmia and ventricular arrhythmia on SLEDAI-2K levels in different subgroup; Table S7: The association of sinus arrhythmia on SLEDAI-2K levels in different subgroup; Table S8: The association of atrioventricular block on SLEDAI-2K levels in different subgroup; Table S9: The association of other kinds of ECG abnormal on SLEDAI-2K levels in different subgroup; Table S10: The association of ST-T change, atrial arrhythmia and ventricular arrhythmia, sinus arrhythmia, strioventricular block, other kinds of ECG abnormal on SLEDAI-2K level.

## Abbreviations and Acronyms

SLE: systemic lupus erythematosus; ECG: electrocardiography; CVD: cardiovascular disease; RCS: restricted cubic spline; LN: lupus nephritis; SLEDAI-2K: Systemic Lupus Erythematosus Disease Activity Index 2000.
