# Supplementary material for: Association Between Disease Activity of Systemic Lupus Erythematosus and Resting Electrocardiogram Abnormalities
Source: J Clin Med. 2025 Mar 7;14(6):1799. doi: 10.3390/jcm14061799 (PMC11943163; doi:10.3390/jcm14061799)
Supplement: Supplementary file 1 [file jcm-14-01799-s001.zip › jcm-3400285-supplementary.pdf]

Supplementary Table S1 Baseline characteristics of the study population by with or without complications or complications

Activity of disease	No complications and comorbidities	Complications and comorbidities	P value
N	155	162	
Gender			0.563
Female	140 (90.3%)	142 (87.7%)	
Male	15 (9.68%)	20 (12.3%)	
Age (year)	30.0 [24.0;44.0]	30.0 [22.2;40.0]	0.263
Age class			0.192
＞25	112 (72.3%)	105 (64.8%)	
≤25	43 (27.7%)	57 (35.2%)	
Disease duration	1.00 [0.00;6.00]	2.00 [0.00;5.00]	0.584
SLEDAI	4.00 [2.00;7.00]	8.00 [4.00;12.8]	**<0.001**
Disease activity			**<0.001**
Inactive	89 (57.4%)	49 (30.2%)	
Mild activity	49 (31.6%)	47 (29.0%)	
Moderate activity	14 (9.03%)	35 (21.6%)	
Severe activity	3 (1.94%)	31 (19.1%)	
Neuropsychiatric SLE			**<0.001**
No	155 (100%)	138 (85.2%)	
Yes	0 (0.00%)	24 (14.8%)	
Lupus nephritis			**<0.001**
No	155 (100%)	63 (38.9%)	
Yes	0 (0.00%)	99 (61.1%)	
Interstitial lung disease			**<0.001**
No	155 (100%)	145 (89.5%)	
Yes	0 (0.00%)	17 (10.5%)	
Hematological involvement			**<0.001**
No	155 (100%)	138 (85.2%)	
Yes	0 (0.00%)	24 (14.8%)	
Combine autoimmune diseases			**<0.001**
No	155 (100%)	121 (74.7%)	
Yes	0 (0.00%)	41 (25.3%)	
Infectious complications			0.920
No	144 (92.9%)	149 (92.0%)	
Yes	11 (7.10%)	13 (8.02%)	

Supplementary table S1 Baseline characteristics of the study population by with or without complications or complications(continue)

Activity of disease	No complications or complications	Complications or complications	P value
Sinus arrhythmia			0.083
No	134 (86.5%)	127 (78.4%)	
Yes	21 (13.5%)	35 (21.6%)	
Atrioventricular block			0.472
No	145 (93.5%)	147 (90.7%)	
Yes	10 (6.45%)	15 (9.26%)	
ST-T change			0.187
No	103 (66.5%)	95 (58.6%)	
Yes	52 (33.5%)	67 (41.4%)	
Other kinds of ECG abnormal			0.971
No	138 (89.0%)	143 (88.3%)	
Yes	17 (11.0%)	19 (11.7%)	
Serum creatinine	54.5 [48.8;65.5]	64.3 [51.6;88.8]	**<0.001**
Complement C 3	0.81 [0.60;1.04]	0.62 [0.38;0.80]	**<0.001**
Complement C 4	0.16 [0.11;0.22]	0.12 [0.06;0.18]	**<0.001**
Anti-nuclear antibodies	320 [100;1000]	1000 [320;2720]	**0.015**
Anti-ds DNA Antibodies	1.00 [0.00;73.0]	43.5 [0.00;92.0]	**0.037**
Anti-RNP antibody	0.00 [0.00;1.00]	1.00 [0.00;1.00]	0.784
Anti-smith Antibodies	0.00 [0.00;1.00]	0.00 [0.00;1.00]	0.761
Anti-SSB antibodies	0.00 [0.00;1.00]	0.00 [0.00;1.00]	0.966
Anti-Jo-1 antibody	0.00 [0.00;0.00]	0.00 [0.00;0.00]	0.409
Anti-Scl-70 antibody	0.00 [0.00;0.00]	0.00 [0.00;0.00]	0.231
Anti-SSA antibody	1.00 [0.00;1.00]	1.00 [0.00;1.00]	0.435

Bold indicates statistical significance

The Student’s t-test was utilized for continuous variables, and the chi-square test was employed for categorical variables to assess differences in the descriptive analyses.

Supplementary Table S2 The association between SLEDAI-2K and the risk of sinus arrhythmia

Sinus arrhythmia	OR（95%CI）		
	Model 1	Model 2	Model 3
SLEDAI(continuous)	1.01(0.96, 1.06)	1.02(0.96, 1.07)	0.98(0.92, 1.05)
	P=0.655	P=0.506	P=0.652
SLEDAI(classification)			
Inactive	Reference	Reference	Reference
Mild activity	0.98(0.47, 1.98)	0.97(0.46, 1.99)	0.79(0.35, 1.74)
	P=0.948	P=0.937	P=0.564
Moderate activity	0.60(0.19, 1.57)	0.60(0.19, 1.60)	0.36(0.10, 1.10)
	P=0.330	P=0.343	P=0.091
Severe activity	1.90(0.75, 4.52)	2.23(0.86, 5.52)	1.52(0.46, 4.89)
	P=0.157	P=0.087	P=0.484

Bold indicates statistical significance

Model 1: No covariates were adjusted

Model 2: Age, and gender were adjusted

Model 3: Gender; age (≤25 vs >25years); disease duration; cumulative SLE manifestations (neuropsychiatric involvement; LN; haematological involvement; infectious complications; combine autoimmune diseases; serum creatinine; serum complement C 3; serum complement C 4; ANA; anti-double-stranded DNA; anti-smith antibodies; anti-SSB antibodies.

were adjusted.

OR odds ratio, 95%CI 95% Confidence Interval

Supplementary Table S3 The association between SLEDAI-2K and the risk of atrioventricular block

Atrioventricular block	OR（95%CI）		
	Model 1	Model 2	Model 3
SLEDAI(continuous)	1.05(0.99, 1.12)	1.06(0.99, 1.13)	1.08(0.99, 1.19)
	P=0.111	P=0.065	P=0.089
SLEDAI(classification)			
Inactive	Reference	Reference	Reference
Mild activity	0.56(0.15, 1.72)	0.63(0.17, 2.00)	0.65(0.16, 2.25)
	P=0.334	P=0.459	P=0.509
Moderate activity	1.45(0.43, 4.33)	1.79(0.52, 5.59)	2.38(0.54, 10.10)
	P=0.515	P=0.325	P=0.241
Severe activity	2.74(0.87, 8.03)	3.23(1.01, 10.15)	4.13(0.90, 19.93)
	P=0.070	**P=0.046**	P=0.068

Bold indicates statistical significance

Model 1: No covariates were adjusted

Model 2: Age, and gender were adjusted

Model 3: Gender; age (≤25 vs >25years); disease duration; cumulative SLE manifestations (neuropsychiatric involvement; LN; haematological involvement; infectious complications; combine autoimmune diseases; serum creatinine; serum complement C 3; serum complement C 4; ANA; anti-double-stranded DNA; anti-smith antibodies; anti-SSB antibodies.

were adjusted.

OR odds ratio, 95%CI 95% Confidence Interval

Supplementary Table S4 The association between SLEDAI-2K and the risk of other kinds of ECG abnormal

Other kinds of ECG abnormal	OR（95%CI）		
	Model 1	Model 2	Model 3
SLEDAI(continuous)	1.03(0.97, 1.08)<br>P=0.375	1.03(0.97, 1.09)<br>P=0.321	1.05(0.97, 1.14)<br>P=0.222
SLEDAI(classification)			
Inactive	Reference	Reference	Reference
Mild activity	1.94(0.87, 4.44)<br>P=0.334	1.92(0.85, 4.42)<br>P=0.118	1.90(0.8, 4.66)<br>P=0.149
Moderate activity	1.19(0.36, 3.42)<br>P=0.515	1.18(0.36, 3.43)<br>P=0.765	1.47(0.39, 4.98)<br>P=0.544
Severe activity	1.4(0.37, 4.35)<br>P=0.070	1.53(0.40, 4.90)<br>P=0.495	1.54(0.28, 7.12)<br>P=0.591

Bold indicates statistical significance

Model 1: No covariates were adjusted

Model 2: Age, and gender were adjusted

Model 3: Gender; age (≤25 vs >25years); disease duration; cumulative SLE manifestations (neuropsychiatric involvement; LN; haematological involvement; infectious complications; combine autoimmune diseases; serum creatinine; serum complement C 3; serum complement C 4; ANA; anti-double-stranded DNA; anti-smith antibodies; anti-SSB antibodies.
were adjusted.

OR odds ratio, 95%CI 95% Confidence Interval

Supplementary Table S5 The association of ST-T change on SLEDAI-2K levels in different subgroup.

Subgroup	OR (95%CI)	P for trend	P for interaction
**Gender**			**0.022**
**Female**	1.05(1.01, 1.10)	**0.014**	
**Male**	1.34(1.13, 1.71)	**0.004**	
**Age class**			0.277
**＞25**	1.06(1.00, 1.12)	0.050	
**≤25**	1.11(1.04, 1.19)	**0.003**	
**Infectious complications**			0.254
**No**	1.08(1.04, 1.13)	**<0.001**	
**Yes**	0.98(0.83, 1.16)	0.828	
**Combine autoimmune diseases**			0.358
**No**	1.09(1.04, 1.14)	**<0.001**	
**Yes**	1.03(0.93, 1.15)	0.565	

OR odds ratio, 95%CI 95% Confidence Interval

Supplementary Table S6 The association of atrial arrhythmia and ventricular arrhythmia on SLEDAI-2K levels in different subgroup.

Subgroup	OR (95%CI)	P for trend	P for interaction
**Gender**			0.438
**Female**	1.03(0.97, 1.08)	0.318	
**Male**	1.09(0.93, 1.27)	0.246	
**Age class**			0.567
**＞25**	1.02(0.95, 1.09)	0.526	
**≤25**	1.05(0.98, 1.13)	0.167	
**Infectious complications**			0.592
**No**	1.04(0.98, 1.09)	0.165	
**Yes**	0.98(0.80, 1.18)	0.852	
**Combine autoimmune diseases**			0.517
**No**	1.04(0.99, 1.10)	0.123	
**Yes**	1.00(0.89, 1.12)	0.987	

Supplementary Table S7 The association of sinus arrhythmia on SLEDAI-2K levels in different subgroup.

Subgroup	OR (95%CI)	P for trend	P for interaction
**Gender**			0.974
**Female**	1.01(0.96, 1.06)	0.700	
**Male**	1.01(0.79, 1.21)	0.945	
**Age class**			0.697
**＞25**	1.01(0.95, 1.08)	0.703	
**≤25**	1.03(0.94, 1.12)	0.425	
**Infectious complications**			0.782
**No**	1.01(0.95, 1.06)	0.784	
**Yes**	1.03(0.86, 1.25)	0.711	
**Combine autoimmune diseases**			0.451
**No**	1.02(0.96, 1.07)	0.535	
**Yes**	0.91(0.63, 1.12)	0.517	

Supplementary Table S8 The association of atrioventricular block on SLEDAI-2K levels in different subgroup.

Subgroup	OR (95%CI)	P for trend	P for interaction
**Gender**			0.911
**Female**	1.05(0.98, 1.13)	0.142	
**Male**	1.06(0.92, 1.23)	0.391	
**Age class**			0.560
**＞25**	1.04(0.95, 1.14)	0.328	
**≤25**	1.09(0.98, 1.20)	0.099	
**Infectious complications**			0.565
**No**	1.05(0.98, 1.11)	0.162	
**Yes**	1.13(0.86, 1.53)	0.350	
**Combine autoimmune diseases**			0.535
**No**	1.06(0.99, 1.14)	0.079	
**Yes**	1.01(0.86, 1.15)	0.847	

Supplementary Table S9 The association of other kinds of ECG abnormal on SLEDAI-2K levels in different subgroup.

Subgroup	OR (95%CI)	P for trend	P for interaction
**Gender**			0.493
**Female**	1.03(0.97, 1.09)	0.318	
**Male**	0.92(0.58, 1.18)	0.606	
**Age class**			0.628
**＞25**	1.03(0.95, 1.10)	0.480	
**≤25**	1.06(0.95, 1.16)	0.245	
**Infectious complications**			0.580
**No**	1.03(0.97, 1.09)	0.299	
**Yes**	0.90(0.46, 1.33)	0.665	
**Combine autoimmune diseases**			0.904
**No**	1.03(0.96, 1.09)	0.384	
**Yes**	1.02(0.87, 1.16)	0.791	

Supplementary Table S10 The association of ST-T change, atrial arrhythmia and ventricular arrhythmia, sinus arrhythmia, strioventricular block, other kinds of ECG abnormal on SLEDAI-2K level

SLEDAI	β (95%CI)		
	Model 1	Model 2	Model 3
ST-T change	0.07(0.03, 0.12)	0.08(0.04, 0.12)	0.09(0.04, 0.15)
	**P<0.001**	**P<0.001**	**P<0.001**
Atrial arrhythmia and ventricular arrhythmia	0.03(-0.02, 0.08)	0.04(-0.01, 0.09)	0.03(-0.03, 0.09)
	P=0.180	P=0.093	P= 0.365
Sinus arrhythmia	0.01(-0.04, 0.06)	0.02(-0.04, 0.07)	-0.02(-0.09, 0.05)
	P=0.655	P=0.506	P=0.652
Atrioventricular block	0.05(-0.02, 0.11)	0.06(-0.01, 0.12)	0.08(-0.01, 0.17)
	P=0.111	P=0.065	P=0.089
Other kinds of ECG abnormal	0.03(-0.03, 0.08)	0.03(-0.03, 0.09)	0.05(-0.03, 0.13)
	Pp=0.375	P=0.321	P=0.222

Bold indicates statistical significance

Model 1: No covariates were adjusted

Model 2: Age, and gender were adjusted

Model 3: Gender; age (≤25 vs >25years); disease duration; cumulative SLE manifestations (neuropsychiatric involvement; LN; haematological involvement; infectious complications; combine autoimmune diseases; serum creatinine; serum complement C 3; serum complement C 4; ANA; anti-double-stranded DNA; anti-smith antibodies; anti-SSB antibodies.

were adjusted.

95%CI 95% Confidence Interval
